# Bright green light treatment of depression for older adults [ISRCTN69400161]

**DOI:** 10.1186/1471-244X-5-42

**Published:** 2005-11-09

**Authors:** Richard T Loving, Daniel F Kripke, Nancy C Knickerbocker, Michael A Grandner

**Affiliations:** 1 Department of Psychiatry, University of California, San Diego, USA

## Abstract

**Background:**

Bright white light has been successfully used for the treatment of depression. There is interest in identifying which spectral colors of light are the most efficient in the treatment of depression. It is theorized that green light could decrease the intensity duration of exposure needed. Late Wake Treatment (LWT), sleep deprivation for the last half of one night, is associated with rapid mood improvement which has been sustained by light treatment. Because spectral responsiveness may differ by age, we examined whether green light would provide efficient antidepressant treatment in an elder age group.

**Methods:**

We contrasted one hour of bright green light (1,200 Lux) and one hour of dim red light placebo (<10 Lux) in a randomized treatment trial with depressed elders. Participants were observed in their homes with mood scales, wrist actigraphy and light monitoring. On the day prior to beginning treatment, the participants self-administered LWT.

**Results:**

The protocol was completed by 33 subjects who were 59 to 80 years old. Mood improved on average 23% for all subjects, but there were no significant statistical differences between treatment and placebo groups. There were negligible adverse reactions to the bright green light, which was well tolerated.

**Conclusion:**

Bright green light was not shown to have an antidepressant effect in the age group of this study, but a larger trial with brighter green light might be of value.

## Background

Bright white light has been shown to suppress melatonin, shift circadian rhythms and alleviate depression. Evidence suggests that green light may have effects similar to those of white light but could be more efficient [1-4]

Recent studies have suggested that lower photon densities of blue light are required to suppress melatonin or to shift circadian phase than green, yellow, or red light [5-8]. It has been suggested that ganglion cells supplying the retinohypothalamic tract may be particularly responsive to blue light because they contain the blue-light-absorbing photopigment, melanopsin [9,10]. Retinohypothalamic transmission is key to light suppression of melatonin and to light-induced circadian phase shifting. However, there are reasons for skepticism that blue light is the best choice for light treatment of depression. The role of the retinohypothalamic tract in the antidepressant effects of bright light has not been fully established. The retinohypothalamic tract can be stimulated by light in the absence of melanopsin, suggesting an ancillary role for rods or cones which may be more sensitive to green light [11]. Indeed, the actual empirical peak wavelength for stimulation of melanopsin cells was about 500 nm (blue-green), although fitting an opsin curve to the data yielded interpolated blue peaks around 470–480 nm [12,13]. A single opsin curve cannot be accurately explanatory if melanopsin, rods, and cones are all involved. In some studies, adjustment of retinal sensitivity spectra for attenuation of blue light by the ocular lens may have suggested a greater advantage for blue light than was empirically observed with corneal illumination [5]. Indeed, in older subjects, whose yellowing lens increasingly attenuates blue light, green light might be as effective as blue light in suppressing melatonin [14-16]. The practical advantage of blue light may also be exaggerated by expressing sensitivity in photon densities, since blue photons contain more energy as compared to green or red. The risks of blue light are greater [17]. For these considerations, the benefits/risks ratio could be better with green light than with blue light, possibly better than with most white light. Previous studies of green light have suggested positive benefits with modest brightness, e.g., 200–2500 lux [4,18,19].

The mood-improving effects of one night of partial sleep deprivation are enhanced by subsequent daily use of light treatment. Our laboratory's earlier studies, [20,21] the Praskos' study, [22] and the work of Neumeister et al., [23] Loving, [24] and Bloching [25] all indicated that Late Wake Treatment (LWT), when combined with bright light, produces remarkable antidepressant responses, demonstrated by dramatic contrasts between bright light and placebo. Considering the evidence that LWT may accentuate the contrast of bright light and placebo, we believed LWT would add to the potential light treatment effect anticipated in this study.

In a previous study to be reported separately, we found no significant benefit of bright white light for elderly depressed outpatients . This study sought to determine if light resistance in the 60–79 year age range could be overcome with bright green light. Specifically, we ran a 4-week clinical trial of morning bright green light versus dim red placebo light, as an adjunctive antidepressant treatment.

Besides changing the active treatment from 8,500 lux white fluorescent light to 1,200 lux green LED light, this study differed from our previous clinical trial of depressed elders by focusing on morning treatment regardless of the subject's chronotype (circadian adjustment) and by randomizing active treatment immediately after intake, without baseline recording. In this study, no hormone data were collected.

## Methods

Recruitment for depressed individuals, age 60–79 years, was conducted by advertising and public presentations, from February 2004 to January 2005. Written informed consent was obtained from each participant in accordance with the guidelines set forth by the Declaration of Helsinki. The study protocol and consent form were approved by the UCSD Human Research Protection Program. In addition, those participants who were being treated for depression by either a physician or counselor were requested to obtain the written agreement of the therapist for the study, to assure that there was no interference with ongoing treatment and treatment responsibility. Patients were encouraged to continue ongoing treatment during the study, with the assumption that psychotherapy and medication effects over an interval of 4 weeks were likely to be small and randomized between groups.

For enrollment in the study, a Geriatric Depression Scale (GDS) score of 11 (indicating probable major depression) [26] was required. Meeting full DSM-IV criteria for current major depressive disorder was not required, because many aging depressed people need treatment without meeting criteria for major depressive disorder [27]. As a history of mania appears to predict a greatly increased risk of a manic switch during bright light treatment, any volunteer with a history of mania was excluded [28]. Volunteers who were outdoors so much that artificial light treatment would add little were also excluded. For example, if they were outdoors for more than an hour at times of potential light triggered circadian rhythm shifts, that is during morning or evening hours. The *Structured Clinical Interview for DSM-IV Axis I Disorders (Non-patient Edition) *[29] was administered during the assessment period.

**Figure 1:**
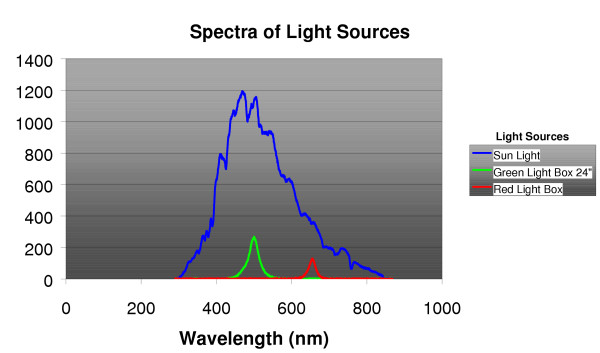
Spectrophotometric measures of illumination are shown comparing daylight with the green and red treatment lights. Sunlight was measured with the photometer pointed towards the horizon (and shaded from direct sun) near noon on a clear sunny day (32.85 North latitude, 2/2/05). Green light was measured at 2 feet with the photometer oriented towards the center of the box. The red light was measured with the photometer adjacent to the diffuser, because at 2 feet, the illumination was too dim to be plotted on the same scale. The irradiance scale was arbitrary (uncalibrated) but identical for the three measures.

Subjects were then randomized into one of two treatment groups: A) 1,200 lux bright green light or B) 10 lux dim-red-light placebo. Computerized randomized assignment within blocks, using sealed envelopes, was stratified for age below or ≥ 68 years, and baseline GDS score below or ≥ 16. One hour of treatment was self-administered within one hour of awakening. Subjects were instructed to place the light box at eye level and to sit so their eyes were 24" from the light box. Both red (SunBox Company, Gaithersburg, MD) and green (Apollo Health, Orem, UT) light boxes were specially built for the study. They each used light emitting diodes (LED) as the light source (See Figure 1). Both light sources were enclosed in standard light treatment boxes, approximately 18" × 24" × 4", with a clear light diffuser panel on the front.

We employed dim red light as a placebo, reasoning that because the light was dim and because the red part of the spectrum may be relatively inactive biologically [5] there would be no substantial biological effect. Fortunately, claims by others of red-light benefits allowed us to tell volunteers, without deception, that some people think that red light is active, even though we are skeptical. In this way, we attempted to keep subjects blind to treatment expectations.

During the four week protocol, the volunteers completed sleep-activity logs daily. They continuously wore an Actillume wrist monitor (Ambulatory Monitoring Inc., Ardsley, N.Y.) to record sleep-wake data and illumination data.

To test the benefits of partial sleep deprivation, on the initial night of the research protocol, we asked volunteers to awaken themselves 4 hours after going to bed and to remain awake for the second half of the night. They were asked to call our telephone answering machine every half hour to confirm that they had been awake for that time period. In a previous study, we found such home sleep deprivations work well without complication [24], but in our previous study of depressed elderly, few could successfully comply.

To assess subject expectations for the two treatments, measures were taken at the beginning and end of treatment, using 100 mm visual analog scales for both mood and sleep improvement. The initial rating was obtained after the subject was randomized and had seen the light they would be using but before the first actual treatment. A final assessment was obtained on the last day of the study.

Four weeks of treatment were carried out with weekly symptom assessments and continuous wrist recordings of activity and illumination exposure. The investigators visited subjects weekly to assure their safety and their compliance with the study, to administer and collect rating forms, and to transfer data from the Actillume recorders. A final symptom and circadian assessment was completed in the last 48 hours of the 4-week randomized treatment. Two-week and 4-week follow-up assessments were obtained.

In addition to daily log sheets used to record activity, sleep behaviors, and visual analog self ratings of mood, the subjects completed a weekly GDS [26] and a Systematic Assessment for Treatment Emergent Events (SAFTEE) [30] symptom scale. GDS ratings were also completed at the usual time of awakening after the half-night sleep deprivation. Further mood measurements were made at baseline and end-of-treatment using the SIGH_SAD_SR, a self-rating form including the Hamilton Depression Rating Scale (HDRS, 17 items used) and atypical items previously shown to be responsive to light treatment [31]. Additionally, when a graduate student was available, a blind HDRS rating interview was obtained at baseline and in the last week of the study.

Records from the Actillume monitor indicating total activity, sleep-wake, and log_10_[lux] were fitted to cosine curves for each subject. The mesors or fitted cosine means were examined, as well as the acrophases which indicate the time of day of the fitted peak.

## Results

Sixty-one potential participants signed consents and were initially screened. Twenty-eight candidates did not meet GDS criteria, and 33 subjects completed the protocol, 5 males and 28 females. Bright green light treatment was received by 17 subjects, and 16 received dim red light placebo. The mean age for those completing the study was 67.7 years (SD = 6.35) and ranged from 59 to 80.

Based on the SCID interviews, DSM IV Axis I diagnoses for the sample were Major Depressive Disorder (N = 30) and Minor Depressive Disorder (N = 1). Two subjects had GDS ≥ 11 but did not meet SCID criteria for any Axis I diagnosis. None of the subjects met criteria for DSM IV Seasonal Trend. The characteristics of these depressed subjects ranged from single episodes to chronic recurrent conditions and varied in severity. This added to the general applicability of the treatments.

Of the 33 subjects who completed the study, 13 were being seen by a psychotherapist during research treatment. Of these 13, 7 received bright green light and 6 received dim light. In all 13 cases, treatment was stable during the study. Antidepressants medication was used by 13 of the subjects (5 bright green light, 8 dim light). Other prescription medications used by participants were; antianxiety (2), cardiac (7), antihypertensive (11), analgesic (6), hypnotic (8), thyroid (9), hormone replacement therapy (9), diabetes drugs (1), cholesterol-lowering compounds (12). Nine subjects received both psychotherapy and psychiatric medication, of whom 5 received bright green light and 4 received dim light. The 5 subjects receiving bright green light had no medication changes, and of the 4 dim light subjects, 2 had minor medication changes, one increased fluoxetine from 20 mg to 40 mg per day at week 2 and one reduced citalopram from 20 mg to 10 mg only for the second week of the study.

For the 33 subjects who completed the protocol, groups assigned to active and control light treatment were balanced in age and severity of depression (see Table 1). Expectations for sleep and mood effects of light treatment are shown in Table 2. A repeated-measures MANOVA as well as a one-way ANOVA of change scores showed no difference in the sleep and mood expectations between the bright and dim treatment groups either before or after treatment, suggesting that the dim red light was an active control which balanced expectations.

**Table 1:** Stratification by Age and Depression Severity

**Age-Depression Severity Group**	**Bright**	**Dim**	**Total**
**Age < 68, GDS < 16**	0	0	0
**Age < 68, GDS ≥ 16**	10	10	20
**Age ≥ 68, GDS < 16**	2	2	4
**Age ≥ 68, GDS ≥ 16**	5	4	9
**Total**	17	16	33

**Table 2:** Expectations for Improvement in Sleep and Mood

100 mm Visual Analog Scale, 0 = Worse 100 = Better								
**Measure**	**Mood**				**Sleep**			

**Light**	**Green**		**Red**		**Green**		**Red**	

**Time**	**Initial**	**Final**	**Initial**	**Final**	**Initial**	**Final**	**Initial**	**Final**

**N**	16	16	14	14	16	16	14	14
**Mean**	76.3	69.6	83.4	70.1	71.7	71.8	78.9	64.1
**SD**	18.0	13.6	12.5	20.0	20.9	9.7	12.9	24.0

### Sleep changes

Sleep parameters were not statistically different between the green light treatment group and the red light placebo group. There was no significant effect on sleep onset time or sleep offset time, nor did total sleep time vary significantly by treatment. The total amount of Actillume-estimated sleep was balanced at baseline and not significantly affected by treatment assignment. The baseline, initial week, estimate of total sleep during the nocturnal period was 332 minutes. During the final week the estimate of total sleep during the nocturnal period was 347 minutes. The sleep efficiencies for the initial week and the final week were 75.2 and 74.5 respectively. Wake after sleep onset did not vary at baseline or at the end of treatment. The number of awakenings during the night and the sleep latency did not vary significantly either at the beginning or end of treatment.

### Mood improvement

Mean mood scores for the different groups at each measurement point are shown in Table 3. Subjects' mood improved under both treatments. The average GDS score improved by 7 points (an average of 23%). There were no significant treatment differences in GDS improvement by ANCOVA. The average HDRS17 (extracted from the self-rated SIGH-SAD-SR) improved by 7 points. Improvements following wake treatment were not statistically significant. There were no significant treatment differences in HDRS17 improvement or atypical scores. Blind HDRS17 ratings, when available, were consistent with the self-rating (HDRS17) scores. Power analysis estimated 80% power to detect a large effect size of 0.51 in either the GDS or self-rated HDRS17.

**Table 3:** GDS and HDRS Scores by Week by Light Condition Mean (SD) N

**Light Condition**	**Bright Light**	**Dim Light**
**Baseline**	20.5 (5.19) 17	20.1 (3.98) 16
**After Wake Treatment**	16.1 (7.33) 17	18.5 (4.81) 16
**Treatment week 1**	19.4 (5.51) 16	19.5 (5.26) 14
**Treatment week 2**	15.5 (7.57) 15	15.6 (5.50) 16
**Treatment week 3**	13.3 (6.72) 16	15.9 (5.92) 16
**Treatment week 4**	12.1 (6.25) 17	14.0 (6.30) 16
**Two-week follow-up**	13.5 (7.53) 17	10.7 (6.89) 11
**Four-week follow-up**	12.4 (7.66) 16	10.3 (7.07) 10
**3-Month follow-up**	11.6 (7.59) 13	15.8 (6.94) 6
**HDRS 17 Self Report – Baseline**	19.1 (4.13) 17	17.0 (4.80) 16
**HDRS 17 Self Report – Final**	11.4 (3.94) 17	10.8 (5.01) 16
**Blind HDRS 17 – Baseline**	18.0 (4.87) 13	17.9 (6.34) 14
**Blind HDRS 17 – Final**	11.4 (5.11) 13	10.5 (6.73) 14

### Adverse reactions

Participants experienced no psychiatric hospitalizations, suicide attempts, or deaths during the study or follow-up. There were no incidents of mania or hypomania during the light treatment or during follow-up.

The weekly SAFTEE physical symptom inventory was examined for adverse reactions to both of the light treatments. Ninety-six individual symptoms were evaluated for change during the light treatment period. To improve the stability of measurement, symptoms were grouped into 17 SAFTEE-defined categories, which were tested with Wilcoxon's Signed Rank Test. The results of these group tests are contained in Table 4. The symptom groups for Eyes, Urination, Muscle/Bone, and Other all improved in the bright green light condition. The symptom groups for Mouth/Teeth, Gynecology, and Muscle Bone improved in the dim red light condition. No symptom groups worsened under either light condition The "Other" category contains a large number of mood related items. There were no significant contrasts in SAFTEE changes between treatments.

**Table 4:** SAFTEE Symptoms, Mean Scores for Beginning and End of Light Treatment with Wilcoxon Signed Ranks Test

**Light Condition**	**Bright**			**Dim**		
**Symptom Category**	**Baseline**	**Post Treatment**	**p**	**Baseline**	**Post Treatment**	**p**

**Head**	5.62	4.94	.078	5.31	5.25	.760
**Eyes**	9.41	7.41	**.009**	10.19	8.38	.212
**Ears**	5.12	5.06	.739	5.94	6.06	.661
**Mouth/Teeth**	8.18	7.53	.754	9.19	8.31	**.043**
**Nose/Throat**	7.35	6.59	.052	7.62	6.81	.283
**Chest**	8.64	8.18	.231	7.81	7.88	.666
**Heart**	2.18	2.53	.063	2.12	2.62	.480
**Stomach/Abdomen**	5.88	5.59	.474	5.38	5.69	.144
**Bowel**	8.35	7.59	.192	9.62	8.62	.305
**Appetite**	6.76	6.47	.475	7.50	6.62	.117
**Urination**	7.71	6.76	**.042**	7.12	7.25	.856
**Gynecology**	3.47	3.71	1.00	5.19	2.25	**.026**
**Genital/Sexual**	5.06	5.35	.799	6.38	4.69	.074
**Muscle/Bone**	7.47	6.00	**.011**	6.25	4.81	**.016**
**Walking/Moving**	9.24	8.12	.065	8.62	8.62	.152
**Scalp/Skin**	5.82	5.76	.887	6.44	5.81	.232
**Other**	33.76	24.12	**.001**	27.81	23.06	.052

### Acrophase changes

The two treatment groups had no significant differences in Actillume-recorded activity, sleep, or light exposure at baseline (see Table 5), that is, they were essentially identical in these measures. The fitted peaks (acrophases) of the 24-hour rhythms of activity, sleep, and light exposure were all later in the last week of treatment than the first week, but no contrasts between the bright green and dim red light treatments were significant (see Table 6).

**Table 5:** Baseline Activity, Light, and Sleep Measures by Light Condition

		**ACTIVITY**		**LIGHT**		**SLEEP**	
		
**Baseline**		**MESOR**	**ACROPHASE HH:MM**	**MESOR**	**ACROPHASE HH:MM**	**MESOR**	**ACROPHASE HH:MM**
**Green**	**Mean**	12.42	14:05	1.08	13:29	.27	02:49
	**N**	16	16	16	16	16	16
	**S.D.**	3.77	02:13	.22	01:26	.06	01:46
**Dim Red**	**Mean**	12.52	14:39	1.03	14:07	.27	03:24
	**N**	16	16	16	16	16	16
	**S.D.**	3.80	01:38	.25	01:33	.05	01:49
**p (ANOVA)**		.939	.425	.488	.233	.775	.373

**Table 6:** Acrophase Changes (minutes) by Light Condition Mean (SD) N

**Acrophase Change (minutes) Baseline minus Final**	**Bright green light**	**Dim red light**	**p (ANOVA)**
**Light**	-34.05 (52.32) 15	-5.02 (53.25) 16	0.14
**Activity**	-49.20 (106.75) 16	-5.35 (54.30) 16	0.15
**Sleep**	-34.63 (59.04) 16	-4.48 (67.75) 15	0.20

Note: Negative change indicates a later (delayed) acrophase at the end of treatment.

Although an objective measure of compliance was not available, examination of the light records from the Actillume data suggested a high degree of compliance with treatment. The self reports of treatment time and duration were almost entirely consistent with instructions. The absence of dropouts and the compliance of participants with wearing Actillumes and completing logs and questionnaires also suggested that compliance with treatment was probably high.

### Green light acceptance

The bright green light boxes were well tolerated by the subjects. There was no need to reduce intensity as there sometimes is with bright white light. There was no complaint of eye strain or discomfort. Some of the subjects who had experience with bright white light either before or after the study expressed a preference for the green light.

## Discussion

Bright green light treatment did not increase symptom complaints in the 60–79 year old age group and caused only negligible adverse effects. Although no statistically significant advantages of the 1,200 lux green light were found, the trend in self-ratings favored the green light, and the effect sizes on self-rating depression scales were comparable to those in many trials of 8+ weeks of antidepressant drugs [32]. Most antidepressants drug trials are planned on a larger scale to achieve significance even with small effect sizes (which may be all that the antidepressant achieves). The current study only had power to detect a large effect, which was not observed.

Our observations of the benefits of wake treatment were comparable to the findings in our previous study, again suggesting that wake therapy might not be effective in this age group. Part of the reason is certainly the difficulty of people in this age group in successfully remaining awake for the required interval. It has also been suggested that sleep deprivation may actually interfere with antidepressant treatment for the elderly, [33] which may possibly explain the lack of success of this trial.

Treatments were balanced by age and severity of depression, validating that the stratified randomization procedures were successful. The balancing of participant expectations and the similarity of blind and self-rated HDRS-17 scores indicate that the difficulties in blinding light treatment probably had no influence on the outcome.

There are several possible explanations for the antidepressant effects found from both of the light treatments in this study. The placebo effect must be considered in all clinical research. There were positive expectations for both treatments for both sleep and mood. The socialization and daily structure provided by the study may have lead to improved mood. Other trials have supported the positive effect of green light, but not in this age group. Yellowing of the lens in some elders may tend to attenuate light of 500 nm, though to a lesser extent than shorter wavelengths [15]. The lack of a more distinct advantage for the green light in this study may have been due to inadequate intensity and/or duration of treatment or partial light resistance in this age group. The failure to demonstrate phase advances in actigraphic measures with the green light would be consistent with inadequate green light intensity, light resistance, or poorer compliance than we have otherwise appreciated.

In this our first study with LED green light treatment, we selected a modest intensity of treatment, considerably less than the green component of sunlight and much less than broad-band visible illumination of sunlight (Figure 1). On the one hand, successful white light treatment has often required much more than 1,200 lux. On the other hand, even 400 lux of green light augmented citalopram treatment in a previous study, [19] though a limitation of that study was a possible confounding of the light effect with an earlier waking time. Since the green light was so well-tolerated, a larger-scale trial with somewhat brighter green light would appear safe and might well demonstrate a significant benefit.

## Conclusion

This trial of moderate bright green light contrasted with dim red light placebo did not demonstrate significant difference in mood, sleep or activity measures in this age group. The trail of Late Wake Treatment similarly, did not produce significant improvement in mood measures.

## Competing interests

The author(s) declare that they have no competing interests.

## Authors' contributions

RTL coordinated and carried out the clinical trial, performed statistical analyses, and drafted the manuscript. DFK conceived and drafted the design, administered and participated in data collection, and participated in statistical analyses and manuscript preparation. NCK carried out subject recruitment and collection and scoring of Actillume recordings and subject questionnaires. MAG constructed a software database and administered blind HDRS ratings. All authors reviewed and approved the final manuscript.

## Pre-publication history

The pre-publication history for this paper can be accessed here:


